# Childhood traumatic experiences and functioning of both neurobiological components of the endogenous stress response system in adult people with eating disorders

**DOI:** 10.1192/j.eurpsy.2021.962

**Published:** 2021-08-13

**Authors:** F. Pellegrino, G. Cascino, E. Barone, A.M. Monteleone

**Affiliations:** 1 Department Of Psychiatry, University of Campania “Luigi Vanvitelli”, Naples, Italy; 2 Department Of Medicine Surgery And Dentistry - Section Of Neuroscience, University of Salerno, Salerno, Italy; 3 Department Of Psychiatry, University of Campania “Luigi Vanvitelli”, Naples, Italy

**Keywords:** childhood trauma, eating disorders, alpha-amylase, stress

## Abstract

**Introduction:**

A large body of literature suggests that childhood trauma exposure is a non-specific risk factor for development of eating disorders (EDs) later in life. One potential mechanism through which early traumatic experiences may increase the risk for EDs is represented by long-lasting changes in the body stress response system.

**Objectives:**

We investigated the activity of the hypothalamus-pituitary-adrenal axis and of the sympathetic nervous system in adult ED patients with or without a history of childhood trauma exposure.

**Methods:**

We recruited 35 women with EDs, admitted to the Eating Disorders Center of the Department of Psychiatry of the University of Naples “Luigi Vanvitelli”. Participants filled in the Childhood Trauma Questionnaire (CTQ), to assess exposure to childhood trauma. They were instructed to collect saliva samples at awakening and after 15, 30 and 60 minutes, in order to measure cortisol levels and salivary alpha-amylase (sAA), a marker of the sympathetic nervous system activity.

**Results:**

According to the CTQ cut-off scores, 21 ED women were classified as maltreated (Mal) participants and 14 women as no-maltreated (noMal) ED participants. Compared to noMal ED women, Mal ED participants showed significantly decreased cortisol awakening response (CAR) and sAA morning secretion.

**Conclusions:**

Present findings confirm that childhood trauma exposure impairs the CAR of adult patients with EDs and show that also the morning secretion of sAA is decreased in childhood maltreated adult ED patients. Therefore, our study shows for the first time a dampening in the basal activity of both components of the endogenous stress response system in childhood maltreated adult ED women.

